# Recurrence rate of different techniques for repair of coarctation of aorta: A 10 years experience

**DOI:** 10.4103/0974-2069.74038

**Published:** 2010

**Authors:** Maziar Gholampour Dehaki, Alireza Alizadeh Ghavidel, Nader Givtaj, Gholamreza Omrani, Shahyad Salehi

**Affiliations:** Department of Cardiovascular Surgery, Rajaee Heart Center, Iran University of Medical Sciences and Health Services, Tehran, Iran; 1 Department of Cardiovascular Surgery, Uromiye University of Medical Sciences and Health Services, Imam Street, Imam Khomeini General Hospital, Uromiye, Iran

**Keywords:** Coarctation of aorta, congenital heart disease, heart failure

## Abstract

**Background and Aim:**

The main goal of this study was to assess the frequency of recurrent coarctation after repair using different surgical methods.

**Methods:**

Surgical results of repairs for coarctation of aorta (Co-A) in 188 patients under the age 14 years who were treated in Rajaee Heart Center, Tehran, Iran, were evaluated retrospectively. The most common methods included patch-graft aortoplasty (59%), resection with end-to-end anastomosis (20.7%) and subclavian flap aortoplasty (SCFA) (16.5%). The remaining patients underwent bypass tube graft and excision with placement of a tube graft. Seventy eight percent had discrete stenosis while 22% had long segment narrowing. The patients were followed for 81.6±32.8 months.

**Results:**

The overall mortality rate was 2.6%. The highest incidence rate of recoarctation was found in the patch-graft aortoplasty group (12.7%) and the lowest was found in SCFA (3.2%). The incidence of recoarctation in long-segment lesions was significantly higher than that in the discrete ones (30% vs. 4%, *P*<0.001). In patients <1 year, the incidence of recoarctation was lower than that in the other age groups.

**Conclusion:**

The patch-graft aortoplasty technique had the highest incidence of recoarctation and SCFA had the lowest rate. Long-segment Co-A had a higher chance of recoarctation. In contrast to some previous reports, the incidence of recoarctation was not higher in the age below 1 year.

## INTRODUCTION

Coarctation of aorta (Co-A) is a congenital narrowing in the upper descending thoracic aorta, adjacent to the site of the ductus arteriosus, which causes a pressure gradient across the narrow segment. It is caused by a shelf or infolding of the aortic media into the lumen. Stenosis is either discrete or long segment. Co-A occurs in about 6.5% of the patients with congenital heart disease (CHD) and is the fifth most common CHD.[1]

If left untreated, almost 90% die before the age of 50 years, half of them before 10 years mostly due to heart failure. One-fourth of the deaths occur between the ages of 14 and 20, and these are secondary to endocarditis, intracranial hemorrhage and aortic rupture. Another 25% of the deaths are between the ages of 20 and 50 years and are caused by heart failure, hypertension and valvar disease.[1,2] It has been shown that without treatment, the mean age of survival is about 35 years and thus it seems likely that early intervention can increase the survival rate. Early complications of surgery include functional problems of the left upper extremity after repair with subclavian flap, paraplegia and chylothorax. Late complications include persistent or recurrent Co-A, hypertension and aneurysmal dilation of the aorta. These complications can lower the quality of life and survival and are dependent, in part, on the age at which the repair is carried out, the technique of repair and type of Co-A. Still, controversy exists about the optimal age of operation, with previous and some recent studies indicating that younger age of repair carries a greater risk of recoarctation.[3] In contrast, some recent studies have shown that repair at younger ages are associated with fewer complications such as hypertension.[1,2,4,5] Also, there is no unanimity about the optimal techniques of repair; some believe that repair using subclavian flap, particularly at younger ages, gives the best results.[1,2,4,5]

This study was designed to assess the incidence of major complications of surgery for Co-A, including hemorrhage, recoarctation, paraplegia and chylothorax with various methods of surgery. Also, the relationship between the age of treated patients and recoarctation was evaluated.

## METHODS

In this retrospective study, all patients with a diagnosis of Co-A below the age of 14 years who underwent surgical repair between 1994 and 2004 were included. Patients with interrupted aortic arch and patients who could not be followed-up were excluded from this cohort. Data including age, sex, type of Co-A, associated anomalies, peak systolic gradient before and after repair, technique of surgery, recurrence, hemorrhage, chylothorax, paraplegia, left arm function, follow-up period, mortality, type of intervention after recurrence and its success were extracted and analyzed. Recurrence was defined as a peak systolic gradient of 25 mmHg or more across the repaired segment.

Diagnosis was confirmed using both echocardiography and cardiac catheterization. After surgical repair, all patients underwent echocardiographic assessment and, if recurrence was suspected, cardiac catheterization was also was performed. Data were analyzed with t-test and chi-square test and the *P* value <0. 05 was considered statistically significant.

## RESULTS

In this retrospective study, the data of surgical repair of Co-A in 188 children aged <14 years, who were operated on in “Shahid Rajaei Heart Center” between 1994 and 2004 was collected. The mean age was 5.4±4.2 years. One hundred and thirty-six patients were male (72.3%) and 127 cases had patent ductus arteriosus. Other cardiac anomalies were found in 50% of the patients and included ventricular septal defect, aortic stenosis, bicuspid aortic valve, mitral stenosis, Shone’s complex, atrial septal defect and hypoplastic aortic arch. Most patients had discrete lesion (78%) and the remaining 40 had long-segment lesion [Table 1]. Procedures included patch repair in 111 patients (59%), excision and end-to-end anastomosis in 39 (20.7%), subclavian flap repair in 31 (16.5%), bypass tube graft in five patients (2.7%) and excision with placement of a tube graft in two (1.1%).

**Table 1:** Preoperative patient characteristics

Age (year), mean±SD	5.4±4.2
Sex (male/female)	136/52
Type of Co-A	
Discrete	148 (78)
Long segment	40 (22)
Pre-op systolic PG (mean±SD)	59±18
Associated anomalies	
PDA	127 (67.5)
VSD	41 (21.8)
AS	38 (20)
BAV	29 (15.4)
MS	12 (6.4)
Shone complex	9 (4.8)
ASD	6 (3.2)
Hypoplastic aortic arch	2 (1.1)

PG: peak gradient; PDA: patent ductus arteriosus; VSD: ventricular septal defect; AS: aortic stenosis; BAV: bicuspid aortic valve; MS: mitral stenosis, Figures in parentheses are in percentage

**Figure 1:**
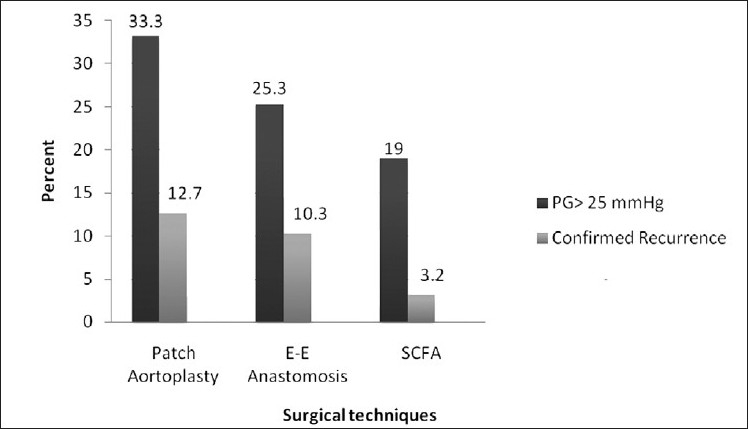
The relative prevalence of recurrence after Co-A repair by various techniques

The follow-up period was 81.6±32.8 months. Recurrence was seen in 54 patients (29%) on echocardiography. Of these, in 19 patients, recoarctation was confirmed at cardiac catheterization 18 of whom, had balloon angioplasty attempted on them. It was successful in 61% of the patients. Only one patient underwent reoperation. The time from surgery to diagnosis of recurrence varied between 0.5 and 8 years, with median of 3.5 years. Of the 111 patients who underwent patch repair, 37 had a peak systolic gradient (PG) ≥25 mmHg on echocardiography and in 14 cases; there was angiographic evidence of recurrence. Of 39 patients with end-to-end anastomosis, 10 had a PG ≥25 mmHg on echo and in four it was confirmed with angiography. In the subclavian flap group (n=31), six had a PG ≥25 mmHg (19%) and only one case presented with angiographic evidence of recoarctation [Figure 1].

A significant reduction of systolic PG was seen in all cases during the early postoperative period. The mean PG decreased from 59±18 mmHg to 18±9 mmHg (*P*<0.01). There were four cases of postoperative surgical bleeding (2.1%) and one case of chylothorax (0.5%). No cases of paraplegia, endocarditis, aneurysm and cerebrovascular accident were found. The overall mortality was 2.6% (5/188). The in hospital mortality rate was 2.1% and one patient died after 2 years due to congestive heart failure. The cause of hospital death was heart failure in three cases and respiratory infection in the other. No case of left arm ischemia or gangrene was detected in the subclavian flap group.

To determine the relationship between age of repair and recurrence, the patients were divided into three groups: <1 year, 1-5 years and 5-14 years. In the first group, 14% had a PG ≥25 mmHg (44% and 29% for groups 2 and 3, respectively) and 4% had documented recurrence on angiography (15% and 10% for groups 2 and 3, respectively). Although there were no significant differences in recurrence between all groups, the lowest recurrence rate was in the first group.

**Figure 2:**
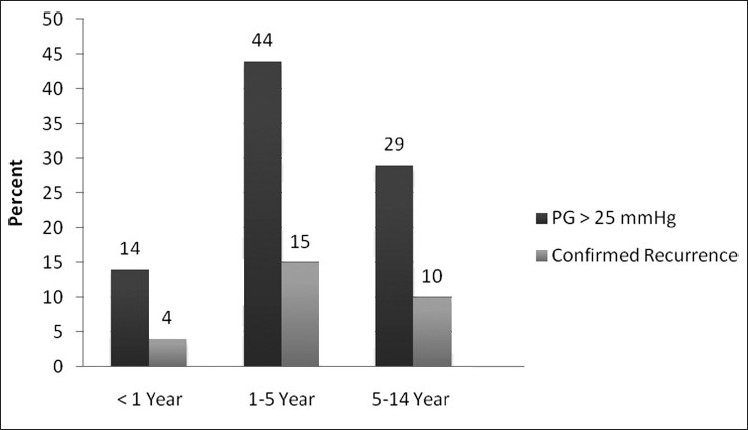
The relative prevalence of recurrence after Co-A repair between age groups

The results are depicted in Figure 2. There were seven (4.7%) recurrences among 148 patients with discrete lesions and 12 (30%) in 40 patients with long-segment lesions [Table 2]. The relationship between cardiac anomalies and recurrence was assessed. Of the patients with PDA (n=127, 67.6%), 30 (23.6%) had a PG≥25 mmHg on echo and eight (6.2%) documented recurrence on angiography. Among patients without PDA (n=61, 32.4%), 24 (39.3%) had a PG ≥25 mmHg on echocardiography and 11 (18%) had recurrence on angiography [Table 3].

**Table 2:** Relationship between type of Co-A and recurrence rate

Type of Co-A	Recurrence rate	
	PG >25 mmHg on echocardiography	Confirmed recurrence on angiography
Discrete (*n*=148, 78%)	*n*=32 (21.6)	*n*=7 (4.7)
Long segment (*n*=40, 22%)	*n*=22 (55)	*n*=12 (30)
*P* value	0.001	0.001

Figures in parenthesis are in percentage

**Table 3:** Relationship between presence of PDA and recurrence

PDA association	Recurrence rate	
	PG >25 mmHg on echocardiography	Confirmed recurrence on angiography
With PDA (*n*=127, 67.6%)	*n*=30 (23.6)	*n*=8 (6.2)
Without PDA (*n*=61, 32.4)	*n*=24 (39.3)	*n*=11 (18)
*P* value	0.030	0.014

Figures in parenthesis are in percentage, PDA: patent ductus arteriosus

## DISCUSSION

In our study, the male/female ratio was 2.6, which was in accordance with the ratio of about 2–3 in the literature.[1–3,6] In this study, the methods of repair were patch repair in 59% of the cases (either Gortex or Dacron), end-to-end anastomosis in 20.7% and subclavian flap repair in 16.5%. The overall incidence of recurrence was 10% in all groups. The recurrence rate reported in different series varied between 5% and 24%.[2,4,7,8] In our study, the angiographically documented recurrence was more prevalent in patch repair (12.7%) and in end-to-end anastomoais (10.3%) than in subclavian flap (3.2%) [Figure 1]. In the series reported by Walhout *et al*, this was about 25%.[7] The recurrence in end-to-end anastomosis repair is reported between 4.2% to 16%.[3,7] In our study, the maximum recurrence was in patch-graft aortoplasty.

In the follow-up period, no left arm acute ischemia, gangrene or functional problems were observed in contrast with the 1% reported ischemia in previous studies. Taking into account the low prevalence of complications and recurrences with the use of subclavian flap, it seems to be advisable for the repair of Co-A at younger ages.

There is no doubt that surgical techniques play a major role in recurrence or persistence of Co-A. For example, inadequate excision of the stenotic segment in the end-to-end anastomosis technique, too much tension on anastomosis, inadequate excision of the intimal shelf and faulty patch and flap play a role in recurrence.

The lowest recurrence rate was among the first group (4%, <1 year), whereas the rate was 15% and 10% for groups 2 and 3, respectively. Uchytil[3] has proposed that repair at younger ages is associated with a greater risk of recurrence. However, recent studies[1,2,4,5,8] have not validated this and, in fact, they have shown that postponing repair can potentially be harmful by lowering the long-term survival and normalization of blood pressure.

## CONCLUSION

Our study has shown least recurrence rate with subclavian flap plasty as compared to patch aortoplasty or end to end anastomosis. There was no relationship between the age of operation and incidence of recurrence. However, long segment coarcts had higher incidence of recurrence as compared to their counterparts with discrete narrowing.
